# Reality of clonidine poisoning in children and adolescents

**DOI:** 10.1111/jpc.16399

**Published:** 2023-04-10

**Authors:** Chi Duong, Caitlyn Lovett, MIchael A Downes, Geoffrey K Isbister

**Affiliations:** ^1^ Emergency Department Angliss Hospital Melbourne Victoria Australia; ^2^ Emergency Department John Hunter Hospital Newcastle New South Wales Australia; ^3^ Department of Clinical Toxicology Calvary Mater Newcastle Newcastle New South Wales Australia; ^4^ Clinical Toxicology Research Group University of Newcastle Newcastle New South Wales Australia

**Keywords:** adolescent, emergency medicine, pharmacology

## Abstract

**Aim:**

We aimed to describe the severity of clonidine poisonings in a paediatric population referred to a tertiary toxicology service.

**Methods:**

We undertook a retrospective review of all presentations of clonidine poisoning in children or adolescents reported to a tertiary toxicology service from March 2014 to February 2020. Cases were divided into young children (0–6 years), older children (7–11 years) and adolescents (12–17 years). We report clinical effects: bradycardia, hypotension and abnormal Glasgow coma score (GCS), based on standard paediatric observation charts, interventions, length of emergency department stay, proportion admitted to a medical ward or paediatric intensive care unit.

**Results:**

We identified 111 clonidine poisonings, 41 young children, 9 older children and 61 adolescents. There were more females in the adolescent group and slightly more males in the younger age groups. The median dose ingested was 13 mcg/kg (interquartile range: 7–38 mcg/kg), which varied across ages. Clonidine alone was ingested in 78 cases (70%) and co‐ingestion was more common in adolescents (24/61; 39%). Thirty‐seven patients (33%) were admitted and 23 (21%) were admitted to paediatric intensive care unit. Median length of emergency department stay was 16.4 h, longer for adolescents. At least one abnormal observation occurred in 101 of 111 (91%) cases: 76 of 106 (72%) bradycardia, 76 of 110 (69%) hypotension and 4 of 99 (4%) GCS < 9. Thirteen (12%) had severe bradycardia, more common in young children and 23 (21%) had severe hypotension, more common in adolescents. For 27 children (0–11 years) ingesting 5–10 mcg/kg, 3 (11%) had severe bradycardia or severe hypotension and 1 received naloxone (4%). No cases ingesting <5 mcg/kg developed moderate/severe bradycardia or hypotension. Four cases received naloxone with no significant change, two patients got atropine with a transient response. One patient was intubated to facilitate safe inter‐hospital transfer.

**Conclusion:**

Paediatric clonidine poisoning commonly results in bradycardia, hypotension and decreased GCS, but rarely severe or requiring major interventions. Children ingesting <5 mcg/kg do not require admission.

## What is already known on this topic

1. There are increasing numbers of paediatric clonidine poisoning cases.
2. Significant resources are used for retrieval and critical care services.

## What this paper adds

1. Despite paediatric clonidine poisoning causing bradycardia, hypotension and central nervous system depression, it is rarely severe.
2. Children ingesting <5 mcg/kg do not require admission.
3. Paediatric clonidine poisoning uncommonly requires critical care admission.

Clonidine is a centrally acting alpha‐2 adrenoreceptor agonist and an imidazoline receptor agonist. These combined actions result in mainly central nervous system and cardiovascular depression.^1^ Although originally introduced as an antihypertensive, it has been superseded by newer agents. It has been used for migraine,^2^ opioid and alcohol withdrawal,^3^ but is now most commonly prescribed in children for behavioural disorders, including attention deficit disorder, tic disorders and sleep disturbance.^3^

Clonidine poisoning has been reported for decades and causes central nervous system depression, hypotension and bradycardia.^2^, ^4^, ^5^ Over the last 3 decades, the epidemiology of clonidine overdose has changed significantly, reflecting the changes in indications and the age of the population it is prescribed in.^6^, ^7^ Early reports were mainly in young children as unintentional ingestions of their parents' medications.^2^, ^7^ There have been increasing adult overdoses^4^ and more recently increasing numbers of deliberate self‐poisoning cases in adolescents.^8^ Cairns *et al*. reported an increase of 4.9% per year of unintentional clonidine poisonings from 2004 to 2017, which correlated with an increase in dispensing in children less than 6 years of age.^9^ In addition, there was an increase in intentional overdoses in children over 11 years of age.^8^

The changing epidemiology of clonidine poisoning means that emergency departments (EDs) and paediatric services are now seeing increasing numbers of cases.^10^ This is despite ingestions <10 mcg/kg in young children with accidental poisoning, not being referred to hospital by Poison centres in Australia. Children presenting with clonidine may be bradycardic with a decreased level of consciousness,^6^ which may prompt concerns from the treating clinician, use of critical care services, including retrieval for intensive care admission and requirement for antidotes, due to the unfamiliarity with this poisoning.^11^

We aimed to describe the severity of clonidine poisonings in a paediatric population referred to a tertiary toxicology service. In addition, we report the interventions and disposition of these patients in a major tertiary children's hospital.

## Methods

### Design and setting

This was a retrospective review of all clonidine poisoning cases aged under 18 reported to a tertiary toxicology service in the Hunter Area of New South Wales. The clinical toxicology service provides inpatient care at a regional tertiary hospital for patients aged 16 years and above as well as covering telephone consultations for 15 EDs within the local health network. This includes telephone advice and joint admission for the regional tertiary paediatric referral centre, which collectively encompasses inpatient paediatric medical and surgical services, a paediatric intensive care unit (PICU) and a paediatric inpatient mental health unit. All telephone consultations to the clinical toxicology service are logged into a tablet‐based database.^12^ The design of the database has been reported in detail previously and was developed for quality assurance purposes.^12^

The toxicology inpatient service is based at an adult facility where there is generally limited data on paediatric poisonings, except direct emergency department presentations that then need to be transferred for paediatric in patient admission. The tablet‐based application allows collection of information on all toxicology consultations within the region,^12^ including all paediatric poisonings, irrespective of the location of presentation.^10^ Clinical data, including demographics, information on the poisoning (toxin, dose and time of ingestion), treatments and outcomes are entered at the time of the consultation into the database by the clinical toxicologist. One record is generated for each patient admission even if there were multiple requests for advice.

### Selection of participants

The database was reviewed for all paediatric clonidine exposures >200 mcg (<18 years), who the toxicology service was consulted about and admitted from 1 March 2014 to 29 February 2020. Enquiries which were outside the referral network as well as those not involving an acute ED presentation were excluded. The cohort was divided into three different age cohorts based on the well‐described epidemiology of paediatric poisoning: 0‐ to 6‐year‐old children, in which unintentional poisoning occurs, 7‐ to 11‐year‐old children, in which poisoning is rare and 12‐ to 17‐year‐old adolescents, in which most cases are deliberate self‐poisoning.^6^, ^10^

### Data collection

Data were extracted from the clinical database, including patient demographics (age and sex), presentation facility (metropolitan, rural), exposure type (accidental ingestion, deliberate self‐poisoning or therapeutic misadventure), source of the clonidine (own, sibling, other family member, friend), details of exposure (dose ingested, co‐ingestants, time of ingestion), interventions (decontamination with activated charcoal, naloxone, intravenous fluids (IVF), intubation, vasoactive pharmacological agents) and disposition. The electronic medical records of admitted patients were also reviewed to correlate and crosscheck data and obtain additional information on vital signs from standard paediatric observation charts (SPOCs; heart rate (HR), blood pressure (BP) and Glasgow coma score (GCS); Fig. S1). Bradycardia and hypotension were defined as normal (white), mild (blue), moderate (yellow) and severe (red) based on the age‐specific SPOCs (Fig. S1).

We included the following outcomes: need for transfer from presenting facility; length of ED admission (LOS) and representation within 48 h; length of stay of admitted patients to a medical ward, mental health unit or PICU, and deaths. We measured LOS for all patients admitted from the time of first presentation in ED until discharge from hospital for that admission.

### Statistical analysis

Medians and interquartile ranges (IQRs) were used to summarise continuous variables and proportions expressed as percentages for categorical variables with 95% confidence intervals. All analyses and graphs were undertaken with GraphPad Prism version 8.2 for Windows (GraphPad Software, San Diego, CA, USA, www.graphpad.com).

The use of the clinical database for research has ongoing approval from the Hunter Area human research ethics committee.

## Results

**Fig. 1:**
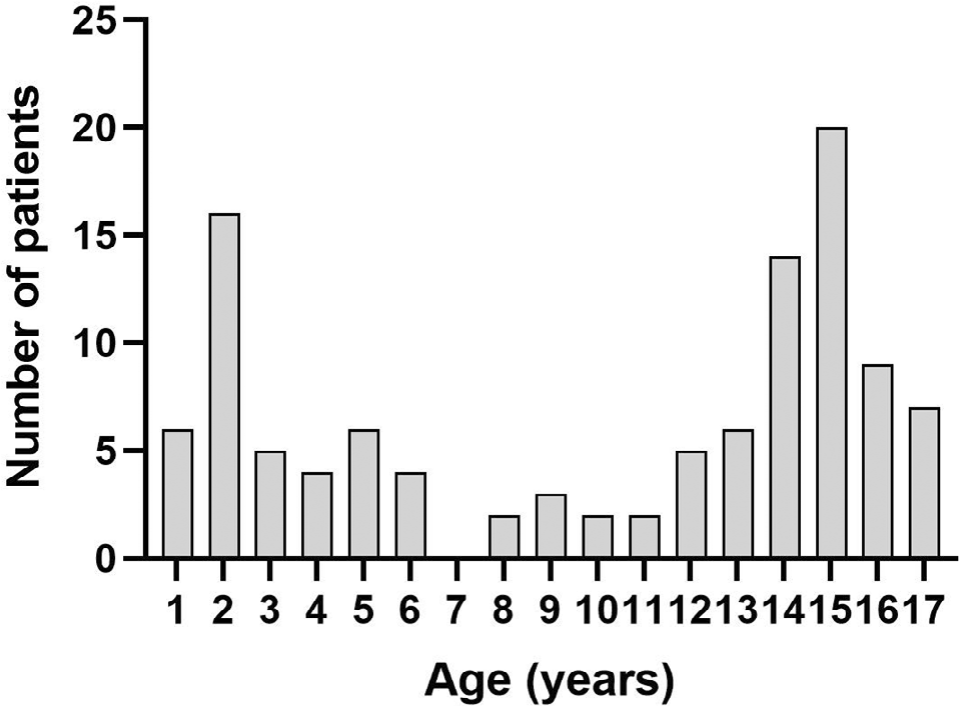
Histogram of the age for all paediatric clonidine poisonings.

There were 123 paediatric clonidine poisonings referred to the toxicology service over the 6‐year period. Ten did not present to hospital or were not transferred to a hospital within the toxicology service referral area. Of the remaining cases, two did not take clonidine and were excluded. The final cohort was 111 clonidine poisonings, including 41 young children aged 0–6 years, 9 older children aged 7–11 years and 61 adolescents aged 12–17 years (Fig. 1). There was a predominance of females in the adolescent group, and slightly more males in the younger two age groups (Table 1). All poisonings in the adolescent group were intentional self‐poisonings, compared with no intentional poisonings in the two younger age groups. Of 61 adolescents, 58 ingested their own medication, compared to only 14 of 41 young children, in which the medication was otherwise sourced from siblings, family or friends (Table 1).

**Table 1:** Characteristics of paediatric patients with clonidine poisoning by age cohort, including demographics, circumstances of the poisoning, dose and disposition

*n* (%)	Total cohort	0–6 years	7–11 years	12–17 years
Number	111	41	9	61
Median age, year	13	2	9	15
Female, *n* (%)	68 (61)	18 (44)	4(44)	46 (75)
Exposure cause, *n* (%)				
Accidental	40 (36)	36 (88)	4 (44)	0
Therapeutic misadventure	10 (9)	5 (12)	5 (56)	0
Deliberate self‐poisoning	61(55)	0	0	61 (100)
Source of clonidine, *n* (%)				
Own medication	79 (71)	14 (34)	7 (78)	58 (95)
Sibling	23 (21)	19 (46)	2 (22)	2 (3)
Other family member	5 (4)	5 (12)	0	0
Friend/family friend	3 (3)	3 (7)	0	0
Unknown	1 (1)	0	0	1 (2)
Presenting location, *n* (%)				
Rural	14 (13)	8 (20)	0	6 (10)
Metropolitan	97 (87)	33 (80)	9 (100)	55 (90)
Dose, mcg/kg, median (IQR)	13 (7–38)	8 (8–26)	6 (6–6)	21 (10–51)
Co‐ingestants, *n* (%)	33 (30)	7 (17)	2 (22)	24 (39)
Disposition, *n* (%)				
Discharged	51 (46)	18 (44)	8 (89)	25 (41)
Ward admission	37 (33)	15 (37)	1 (11)	21 (34)
ICU admission	23 (21)	8 (19)	0	15 (25)
LOS, h, median (range)	16.4 (0.9–708)	12.8 (1.4–51)	3.8 (0.9–14)	19 (2.5–708)

The median dose of clonidine ingested for the total cohort was 13 mcg/kg (IQR: 7–38 mcg/kg), which varied across ages with a median of 8 mcg/kg (IQR: 8–26 mcg/kg) in the 0–6‐year‐old group, 6 mcg/kg (IQR 6–6 mcg/kg) in the 7–11‐year‐old group and 21 mcg/kg (IQR 10–51 mcg/kg) in the adolescent group.

Clonidine alone was ingested in 78 poisonings (70%). Co‐ingestion was more common in the adolescent group, with 24 of 61 (39%) co‐ingesting other medications (Table S1). The majority reported a single co‐ingestant, most commonly paracetamol/anti‐inflammatories, selective serotonin reuptake inhibitors and antipsychotics (Table S1). In the younger age groups most co‐ingestants were methylphenidate^6^ or other psychotropics.^3^

Overall, 60 patients (54%) were admitted to hospital, including 23 (21%) to the PICU. This was similar for all ages, except the 7–11‐year group, in which eight of nine (89%) were discharged home (Table 2). The median LOS was 16.4 h and was longer in the oldest age group (Table 1). There were no deaths.

**Table 2:** Demographics, clinical effects and treatment for unintentional poisoning in children (<12 years old) grouped by mcg/kg

	<5 mcg/kg	5–10 mcg/kg	>10 mcg/kg
Number of cases	5	22	20
Age, median (range), years	4 (1–9)	4 (1–11)	2.5 (1–8)
Female, *n* (%)	3 (60)	11 (50)	7 (35)
Dose, median (range), mcg/kg	4.3 (3.75–4.55)	7.17 (5–9.35)	27.5 (10–500)
Minimum heart rate zone, *n* (%)			
Normal (white)	2 (40)	9 (41)	3 (15)
Mild (blue)	3 (60)	6 (27)	2 (10)
Moderate (yellow)	0	5 (23)	7 (35)
Severe (red)	0	2 (9)	8 (40)
Minimum blood pressure zone, *n* (%)			
Normal (white)	2 (40)	12 (55)	3 (15)
Mild (blue)	3 (60)	2 (9)	6 (30)
Moderate (yellow)	0	6 (27)	8 (40)
Severe (red)	0	2 (9)	3 (15)
Lowest GCS, median (range)	15 (11–15)	15 (3–15)	12 (7–15)
Discharged	4 (80)	15 (68)	6 (30)
Management, *n* (%)			
Observation only	4 (80)	18 (82)	10 (50)
Intravenous fluids	1 (20)	4 (18)	10 (50)
Naloxone	0	1 (5)	2 (10)
SDAC	0	1 (5)	0

GCS, Glasgow coma score; SDAC, single dose activated charcoal.

**Fig. 2:**
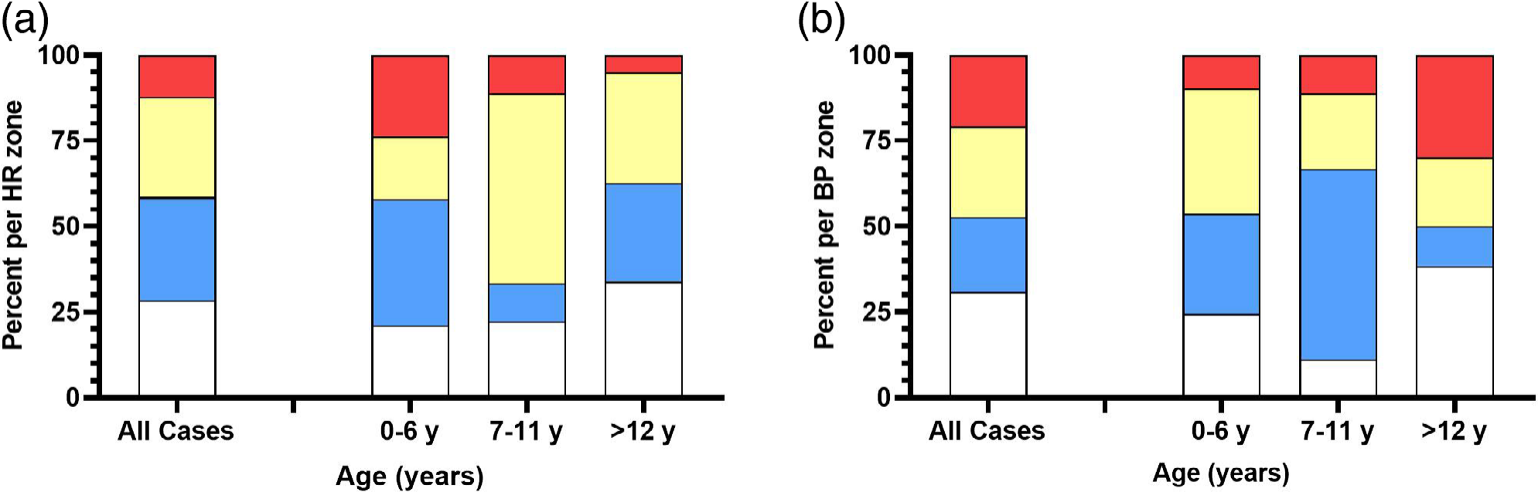
Bar graphs of the heart rate (HR) (a) and blood pressure (BP) (b) divided into normal (white), mild (blue), moderate (yellow) and severe (red) based on the standard paediatric observation charts.

At least one abnormal observation was recorded in 101 of 111 (91%) cases: 76 of 106 (72%) with bradycardia, 76 of 110 (69%) with hypotension and 50 of 99 (51%) with an abnormal GCS. Only 4 of the 99 (4%) had a GCS < 9, 3 in the 0–6‐year group and 1 in the adolescent group. Of the 106 with bradycardia, 31 (29%) had moderate bradycardia (yellow zone) and 13 (12%) had severe bradycardia (red zone). Severe bradycardia was more common in the 0–6‐year group (24%), compared to the adolescent group (5%; Fig. 2). Of the 76 with hypotension, 29 (26%) had moderate hypotension (yellow zone) and 23 (21%) had severe hypotension (red zone), which was more common for the adolescent group (30%) compared to the 0–6‐year group (10%; Fig. 2).

There were 27 cases in which <10 mcg/kg clonidine was accidentally ingested in the two younger age groups (0–11 years; Table 2). While the majority of these had a normal HR and BP (white zone) or mild bradycardia/hypotension (blue zone), five (19%) had moderate bradycardia and two (7%) had severe bradycardia in those who had ingested 5–10 mcg/kg. Similarly, for the 5–10 mcg/kg group, six (22%) had moderate hypotension and two (7%) had severe hypotension. An 11‐year‐old boy ingesting 8 mcg/kg had severe bradycardia and severe hypotension with a HR 57 bpm and BP of 78/53 mmHg, but had no interventions. A 2‐year‐old female ingesting 5 mcg/kg had a HR 60 bpm and a GCS of 11 and was given naloxone with minimal effect. A 5‐year‐old female ingesting 9 mcg/kg had a BP of 85/47 mmHg and GCS of 3 and was admitted to PICU, but had no interventions. There were no cases in the <5 mcg/kg who had moderate or severe bradycardia or hypotension, and only one (20%) received IVF (Table 2).

All cases that were discharged from the emergency department had observation only. For the 37 cases admitted to the ward, 1 received activated charcoal (2 years), 17 IVF and 1 naloxone (Table 3). Of the 23 cases admitted to the PICU one case was intubated to facilitate safe inter‐hospital transfer by the retrieval team. Two cases received an atropine bolus with minimal response. A further case transferred from a peripheral hospital had an adrenaline infusion commenced prior to transfer, which was weaned and ceased in the PICU. Three received naloxone. In all four cases administered naloxone, there were no signs of airway compromise or significant cardiovascular compromise prior to naloxone, and no clinically significant change in the observations was reported (Table S2).

**Table 3:** Treatment for all admissions and whether they were admitted to the general paediatric ward or to the paediatric intensive care unit (PICU)

Treatment	Total cohort (*n* = 60), *n* (%)	Ward admissions (*n* = 37), *n*	PICU admissions (*n* = 23), *n*
Observation only	20 (33)	16	4
Intravenous fluids	40 (67)	20	20
Intubation	1 (1.7)	0	1
Atropine	2 (3)	0	2
Naloxone	3 (5)	1	3
Adrenaline	1 (1.7)	0	1

## Discussion

We found in a large number of paediatric clonidine poisonings the usual bimodal age distribution for paediatric poisoning (Fig. 1). In the adolescent group clonidine exposures were deliberate self‐poisonings of their own medication, with a female predominance and more commonly drug co‐ingestion. Most patients had evidence of toxicity with at least one abnormal observation in 91% of cases. However, only a small number had any intervention, apart from observation and IVF. There were a significant number of younger children (<12 years) ingesting <10 mcg/kg. We found in this group, those who had consumed <5 mcg/kg had minimal toxicity, but those ingesting >5 but <10 mcg/kg had a HR or BP that was in the yellow or red zones on the SPOCs. This suggests that perhaps a new safety dose for paediatric non‐intentional ingestion should be <5 mcg/kg to delineate between those requiring hospital versus home observation. Almost half of young children ingested clonidine sourced from a sibling, supporting the importance of safe medication storage for all children.

The proportion of intentional self‐poisonings in the adolescent group (100%) was consistent with adolescent poisoning in general,^10^ though higher than previously reported in a study by Amico *et al*. in 2019 of 62%.^8^ The median dose in this group was also much higher than for the younger group with exclusively accidental ingestions (21 vs. 8 mcg/kg). Amico *et al*. also reported a similar difference with a doubling of the median dose in the same age groups (0.2 mg vs. 0.5 mg).^8^ Despite the significantly larger median dose in adolescent deliberate self‐poisoning, the severity of poisoning was similar to the younger children, based on abnormal observations and admissions to ICU. Adolescents were more likely to be hypotensive, while young children (0–6 years) were more likely to be bradycardic.

Bradycardia was the most common abnormal effect in 91% of patients with clinical effects. This differed from Cairns *et al*. in which bradycardia occurred in 12.8%, and drowsiness in 44.6% of their patient cohort.^9^ Despite the large proportion of patients who had abnormal observations, less had a HR, BP or GCS in the yellow and red zones on the SPOCs, and even fewer required treatment other than IVF and observation. Two patients were given charcoal, two patients were given atropine, one was administered an adrenaline infusion and one was intubated for interhospital transport. Four patients were given naloxone. This highlights that despite the concerning numbers of cases with drowsiness, hypotension and bradycardia, the overwhelming majority of patients require minimal intervention.

Currently, the Poisons Centre uses a dose of <10 mcg/kg as the ‘safe to stay at home’ limit for non‐intentional ingestions. However, our study demonstrated that only children (<12 years) that ingested <5 mcg/kg, did not have any observations in either the yellow or red zones. This indicates that a more appropriate safety dose for non‐intentional ingestion and close observation at home should be reduced for those patients who have ingested <5 mcg/kg.

In only four patients was naloxone administered. None of the patients who received naloxone had signs of airway or significant cardiovascular compromise prior to administration. There was no clinically significant change in their observations post‐naloxone dose. This contrasts with Seger *et al*., who reported an improvement in drowsiness in 40 of 51 patients.^11^ Due to the small sample size it would be difficult to extrapolate or generalise the significance of our findings, but it does not support the routine use of naloxone.

The main limitations with our study included the fact that serum clonidine concentrations were not measured and the weight of some patients were estimated, both of which can either underestimate or overestimate the median amount of clonidine ingested. Additionally, this was a retrospective study with the potential for bias in reporting observations, especially GCS, but not other objective observations (i.e. HR and BP), and the effects of any interventions.

## Conclusion

We found that although the majority of children and adolescents with clonidine poisoning develop one or more of bradycardia, hypotension or drowsiness, very few require intervention and can be managed with observation and IVF. Children accidentally ingesting >5 mcg/kg should be observed in hospital.

## Supporting information


**Table S1.** Co‐ingested substances in the adolescent (12–17 year old) group.
**Table S2.** Details of the patients who received naloxone treatment and the reported outcome of the treatment.

**Figure S1.** (a) SPOC 1‐4 years. (b) SPOC 5‐11 years. (c) SPOC 12 years and over.
