# Supplementary material for: Reality of clonidine poisoning in children and adolescents
Source: J Paediatr Child Health. 2023 Apr 10;59(6):827–32. doi: 10.1111/jpc.16399 (PMC10946816; doi:10.1111/jpc.16399)
Supplement: Supplementary file 1 — Table S1. Co‐ingested substances in the adolescent (12–17 year old) group. Table S2. Details of the patients who received naloxone treatment and the reported outcome of the treatment. [file JPC-59-827-s001.docx]

**Supplementary File**

**Supplementary Table 1:** Co-ingested substances in the adolescent (12-17 year old) group.

| **Co-ingestant** | **Number** |
| --- | --- |
| Paracetamol/NSAID | 11 |
| SSRI | 10 |
| Psychotropic | 8 |
| Methylphenidate/other ADHD medications | 8 |
| Antibiotics (Amoxycillin/clavulanic acid ) | 2 |
| Melatonin | 2 |
| Valproate, mirtazepine, promethazine, benzodiazepine, hydralazine, coloxyl with senna | 1 |

**Supplementary Table 2:** Details of the patients who received naloxone treatment and the reported outcome of the treatment.

| Case details | Clonidine dose | Signs pre-naloxone | Treatment | Sustained effect |
| --- | --- | --- | --- | --- |
| 2y | 5mcg/kg | GCS 11/15, HR red zone, BP blue zone | 10mg naloxone IV with IV fluid bolus | No – all observations back in same zones within 1 hour. |
| 4y | 125mcg/kg | GCS 10/15, HR in red zone, BP normal | 400mcg IV naloxone | No effect |
| 1.5y | 64mcg/kg | CGS 15/15, HR blue zone, BP red zone | 2mg IV naloxone with IV fluid bolus | Unsustained BP increase |
| 13y | 22mcg/kg | GCS 14/15  HR yellow zone  BP yellow zone | 400mcg IV naloxone  3 hours later, given 300mcg IV atropine (HR red zone, no change) | Unsustained BP increase into white zone |
