# Supplementary material for: Reality of clonidine poisoning in children and adolescents
Source: J Paediatr Child Health. 2023 Apr 10;59(6):827–32. doi: 10.1111/jpc.16399 (PMC10946816; doi:10.1111/jpc.16399)
Supplement: Supplementary file 2 — Figure S1. (a) SPOC 1‐4 years. (b) SPOC 5‐11 years. (c) SPOC 12 years and over. [file JPC-59-827-s002.pdf]

SMR110017

Holes punched as per AS2828.1:2012

BINDING MARGIN - NO WRITING

NSW GOVERNMENT Health

STANDARD PAEDIATRIC OBSERVATION CHART (SPOC)

1 - 4 Years

☐ Altered Calling Criteria

ALL OBSERVATIONS MUST BE GRAPHED

FAMILY NAME	MRN
GIVEN NAME	☐ MALE ☐ FEMALE
D.O.B. ______/______/______	M.O.
ADDRESS	
LOCATION	

COMPLETE ALL DETAILS OR AFFIX PATIENT LABEL HERE

1-4 Years

	Date	Date
	Time	Time
AIRWAY / BREATHING — Respiratory Rate ● (breaths per minute)	80, 75, 70, 65, 60, 55, 50, 45, 40, 35, 30, 25, 20, 15, 10, 5	80, 75, 70, 65, 60, 55, 50, 45, 40, 35, 30, 25, 20, 15, 10, 5
Respiratory Distress	Severe, Moderate, Mild, Normal	Severe, Mod, Mild, Normal
SpO_2 % ●	100, 95, 90, 85, 80, 75, <70, Probe Change	100, 95, 90, 85, 80, 75, <70, Probe Change
Oxygen	L/min or %, Device	L/min or %, Device
CIRCULATION — Heart Rate ● (beats per minute)	220, 210, 200, 190, 180, 170, 160, 150, 140, 130, 120, 110, 100, 90, 80, 70, 60	220, 210, 200, 190, 180, 170, 160, 150, 140, 130, 120, 110, 100, 90, 80, 70, 60
Capillary Refill	≥ 3 Seconds, < 3 Seconds	≥ 3 Seconds, < 3 Seconds
Blood Pressure (mmHg) ˅ ˄ SBP is the trigger	150, 140, 130, 120, 110, 100, 90, 80, 70, 60, 50, 40, 30, 20, 10	150, 140, 130, 120, 110, 100, 90, 80, 70, 60, 50, 40, 30, 20, 10
	Initials	Initials

☐ Increase Frequency of Observations ☐ Clinical Review ☐ Rapid Response

NH606542 201213

NSW GOVERNMENT Health

STANDARD PAEDIATRIC OBSERVATION CHART (SPOC)

1 - 4 Years

☐ Altered Calling Criteria

ALL OBSERVATIONS MUST BE GRAPHED

FAMILY NAME | MRN | GIVEN NAME | ☐ MALE ☐ FEMALE | D.O.B. ______/______/______ | M.O. | ADDRESS | LOCATION

COMPLETE ALL DETAILS OR AFFIX PATIENT LABEL HERE

1-4 Years

DO NOT PRINT

	Date	Date
	Time	Time
DISABILITY — Level of Consciousness	Alert, Verbal, Pain, Unresponsive	Alert, Verbal, Pain, Unresponsive
	Enter appropriate letter. A= Alert, V= Rousable only by voice (consider GCS). P= Rousable only by central pain (conduct GCS). U=Unresponsive	
Pain Score	Severe (7-10), Moderate (4-6), Mild (1-3), Nil	Severe (7-10), Moderate (4-6), Mild (1-3), Nil
EXPOSURE — Temperature (°C) ● (Check unit policy)	41, 40.5, 40, 39.5, 39, 38.5, 38, 37.5, 37, 36.5, 36, 35.5, 35, 34.5, 34	41, 40.5, 40, 39.5, 39, 38.5, 38, 37.5, 37, 36.5, 36, 35.5, 35, 34.5, 34
	BGL	BGL
	Weight	Weight
	Initials	Initials

CONSIDER EARLIER ESCALATION OF PATIENTS WITH

- Chronic or complex conditions
- Post-operative
- Pre-Existing cardiac or respiratory conditions
- Opioid Infusions

ADDITIONAL CRITERIA FOR ESCALATION ON BACK PAGE

ASSESSMENT OF RESPIRATORY DISTRESS

	MILD	MODERATE	SEVERE
Airway	• Stridor on exertion	• Stridor at rest<br>• Partial airway obstruction	• New onset of stridor<br>• Imminent airway obstruction
Behaviour & Feeding	• Normal<br>• Talks in sentences	• Some / intermittent irritability<br>• Difficulty talking or crying<br>• Difficulty feeding or eating	• Agitated / confused<br>• Drowsy<br>• Unable to talk or cry<br>• Unable to feed or eat
Respiratory Rate	• Mildly increased	• Respiratory rate in the Yellow Zone	• Respiratory rate in the Red Zone<br>• Decreasing (exhaustion)
Accessory Muscle Use	• None / minimal	• Moderate recession<br>• Tracheal tug<br>• Nasal flaring	• Severe recession<br>• Gasping<br>• Grunting<br>• Extreme pallor<br>• Cyanosis<br>• Absent breath sounds
Apnoeic Episodes	• None	• Abnormal pauses in breathing	• Apnoeic episodes
Oxygen	• No oxygen requirement	• Mild hypoxaemia, corrected by oxygen<br>• Increasing oxygen requirement	• Hypoxaemia, may not be corrected by oxygen

NSW GOVERNMENT | Health

STANDARD PAEDIATRIC OBSERVATION CHART (SPOC)

1 - 4 Years

☐ Altered Calling Criteria

ALL OBSERVATIONS MUST BE GRAPHED

FAMILY NAME	MRN
GIVEN NAME	☐ MALE ☐ FEMALE
D.O.B. _______ / _______ / _______	M.O.
ADDRESS	
LOCATION	

COMPLETE ALL DETAILS OR AFFIX PATIENT LABEL HERE

1-4 Years

OTHER CHARTS IN USE

☐ **Fluid Balance** ☐ **Insulin Infusion** ☐ **Other** ____________
☐ **Neurological Observation** ☐ **Pain / Epidural / Patient Control Analgesia** ☐ **Other** ____________
☐ **Neurovascular** ☐ **Resuscitation Plan** ☐ **Other** ____________

PRESCRIBED FREQUENCY OF OBSERVATIONS

Observations must be performed routinely at least 4th hourly, unless advised below

**DATE:**	dd/MM/yy				
**TIME:**	hh:mm				
**Frequency Required**	Twice daily				
**Medical Officer Name** (BLOCK letters)	P. SMITH				
**Medical Officer Signature**	P. SMITH				
**Attending Medical Officer Signature**	R. Bloggs				

EXAMPLE

ALTERATIONS TO CALLING CRITERIA
MUST BE REVIEWED WITHIN 48 HOURS OR EARLIER IF CLINICALLY INDICATED
Any alterations MUST be signed by a Medical Officer and confirmed by Attending Medical Officer
Document rationale for altering CALLING CRITERIA in the patient's health care record

		**DATE:**	dd/MM/yy			
		**TIME:**	hh:mm			
		**Next review due Date & Time**	dd/MM/yy hh:mm			
**Vital Sign**	**Zone**	**Standard Thresholds**				
**Respiratory Rate**	Yellow Zone	15 - 20<br>50 - 60				
	Red Zone	<15<br>>60				
**SpO_2**	Yellow Zone	90 - 95				
	Red Zone	<90				
**Heart Rate**	Yellow Zone	70 - 80<br>150 - 170	xxx-xxx			
	Red Zone	<70<br>>170	≤ or ≥ xxx			
**Other**	Yellow Zone					
	Red Zone					
		**Medical Officer Name** (BLOCK letters)	P. SMITH			
		**Medical Officer Signature**	P. SMITH			
		**Attending Medical Officer Signature**	R. Bloggs			

EXAMPLE

	Date	Time	INTERVENTIONS / COMMENTS / ACTIONS
**1.**			
**2.**			
**3.**			
**4.**			

REFER TO YOUR LOCAL CLINICAL EMERGENCY RESPONSE SYSTEM (CERS) PROTOCOL FOR INSTRUCTIONS ON HOW TO MAKE A CALL TO ESCALATE CARE FOR YOUR PATIENT

CHECK THE HEALTH CARE RECORD FOR AN END OF LIFE CARE PLAN WHICH MAY ALTER THE MANAGEMENT OF YOUR PATIENT

Blue Zone Response

IF YOUR PATIENT HAS ANY BLUE ZONE OBSERVATIONS YOU MUST

1. Initiate appropriate clinical care
2. Increase the frequency of observations, as indicated by your patient's condition
3. Manage anxiety, pain and review oxygenation in consultation with the **NURSE IN CHARGE**
4. You can make a call to escalate the care of your patient at any time if you are worried or unsure whether to call

Consider the following:

1. What is usual for your patient and are there documented 'ALTERATIONS TO CALLING CRITERIA'?
2. Does the abnormal observation reflect deterioration in your patient?
3. Is there an adverse trend in observations?

Yellow Zone Response

IF YOUR PATIENT HAS ANY YELLOW ZONE OBSERVATIONS OR ADDITIONAL CRITERIA* YOU MUST

1. Initiate appropriate clinical care
2. Repeat and increase the frequency of observations, as indicated by your patient's condition
3. Consult promptly with the **NURSE IN CHARGE** to decide whether a **CLINICAL REVIEW** (or other CERS) call should be made

Consider the following:

- What is usual for your patient and are there documented 'ALTERATIONS TO CALLING CRITERIA'?
- Does the trend in observations suggest deterioration?
- Is there more than one Yellow Zone observation or additional criteria?
- Are you concerned about your patient?

IF A CLINICAL REVIEW IS CALLED:

1. Reassess your patient and escalate according to your local CERS if the call is not attended within 30 minutes or you are becoming more concerned
2. Document an A-G assessment, reason for escalation, treatment and outcome in your patient's health care record
3. Inform the Attending Medical Officer that a call was made as soon as is practicable

***Additional YELLOW ZONE Criteria**

- Increasing oxygen requirement
- Poor peripheral circulation
- Greater than expected fluid loss
- Reduced urine output or anuria (< 1mL/kg/hr)
- Altered mental state: Agitation, Combative or Inconsolable
- New, increasing or uncontrolled pain
- New onset of fever > 38.5°C
- BGL 2-3mmol/L
- **Concern by you or any staff or family member**

CONSIDER IF YOUR PATIENT'S DETERIORATION COULD BE DUE TO SEPSIS, DEHYDRATION / HYPOVOLAEMIA / HAEMORRHAGE, OR AN OVERDOSE / OVER SEDATION

Red Zone Response

IF YOUR PATIENT HAS ANY RED ZONE OBSERVATIONS OR ADDITIONAL CRITERIA# YOU MUST CALL FOR A RAPID RESPONSE (as per local CERS) AND

1. Initiate appropriate clinical care
2. Inform the **NURSE IN CHARGE** that you have called for a Rapid Response
3. Repeat and increase the frequency of observations, as indicated by your patient's condition
4. Document an A-G assessment, reason for escalation, treatment and outcome in your patient's health care record
5. Inform the Attending Medical Officer that a call was made as soon as is practicable

#Additional RED ZONE Criteria

- **Cardiac or respiratory arrest**
- **Circulatory collapse**
- **Patient unresponsive**
- **New onset of stridor**
- Deterioration not reversed within 1 hour of Clinical Review
- 3 or more simultaneous 'Yellow Zone' observations
- Significant bleeding
- Sudden decrease in Level of Consciousness (a drop of 2 or more points on the GCS)
- New or prolonged seizure activity
- BGL < 2mmol/L or symptomatic
- Lactate ≥ 4mmol/L
- **Serious concern by you or any staff or family member**

DO NOT PRINT

BINDING MARGIN - NO WRITING

Holes punched as per AS2828.1:2012

SMR110019

BINDING MARGIN - NO WRITING

Holes Punched as per AS2828.1: 2012

NSW GOVERNMENT | Health

STANDARD PAEDIATRIC OBSERVATION CHART (SPOC)

12 Years and over

☐ Altered Calling Criteria

ALL OBSERVATIONS MUST BE GRAPHED

FAMILY NAME	MRN
GIVEN NAME	☐ MALE ☐ FEMALE
D.O.B. ____/____/____	M.O.
ADDRESS	
LOCATION	

COMPLETE ALL DETAILS OR AFFIX PATIENT LABEL HERE

Date / Time

AIRWAY / BREATHING

Respiratory Rate ● (breaths per minute): 60, 55, 50, 45, 40, 35, 30, 25, 20, 15, 10, 5

Respiratory Distress: Severe, Moderate (Mod), Mild, Normal

SpO_2 % ●: 100, 95, 90, 85, 80, 75, <70, Probe Change

Oxygen: L/min or %, Device

CIRCULATION

Heart Rate ● (beats per minute): 180, 170, 160, 150, 140, 130, 120, 110, 100, 90, 80, 70, 60, 50, 40

Capillary Refill: ≥ 3 Seconds, < 3 Seconds

Blood Pressure (mmHg) ∨ ∧ SBP is the trigger: 200, 190, 180, 170, 160, 150, 140, 130, 120, 110, 100, 90, 80, 70, 60, 50, 40, 30, 20

Initials

Increase Frequency of Observations | Clinical Review | Rapid Response

NH606544 201213

NSW GOVERNMENT | Health

STANDARD PAEDIATRIC OBSERVATION CHART (SPOC)

12 Years and over

☐ Altered Calling Criteria

ALL OBSERVATIONS MUST BE GRAPHED

FAMILY NAME	MRN
GIVEN NAME	☐ MALE ☐ FEMALE
D.O.B. ____/____/____	M.O.
ADDRESS	
LOCATION	

COMPLETE ALL DETAILS OR AFFIX PATIENT LABEL HERE

Date / Time

DISABILITY

Level of Consciousness: Alert, Verbal, Pain, Unresponsive

Enter appropriate letter. A= Alert, V= Rousable only by voice (consider GCS). P= Rousable only by central pain (conduct GCS). U=Unresponsive

Pain Score: Severe (7-10), Moderate (4-6), Mild (1-3), Nil

EXPOSURE

Temperature (°C) ● (Check unit policy): 41, 40.5, 40, 39.5, 39, 38.5, 38, 37.5, 37, 36.5, 36, 35.5, 35, 34.5, 34

BGL

Weight

Initials

CONSIDER EARLIER ESCALATION OF PATIENTS WITH

- Chronic or complex conditions
- Post-operative
- Pre-Existing cardiac or respiratory conditions
- Opioid Infusions

ADDITIONAL CRITERIA FOR ESCALATION ON BACK PAGE

ASSESSMENT OF RESPIRATORY DISTRESS

	MILD	MODERATE	SEVERE
Airway	• Stridor on exertion	• Stridor at rest<br>• Partial airway obstruction	• New onset of stridor<br>• Imminent airway obstruction
Behaviour & Feeding	• Normal<br>• Talks in sentences	• Some / intermittent irritability<br>• Difficulty talking or crying<br>• Difficulty feeding or eating	• Agitated / confused<br>• Drowsy<br>• Unable to talk or cry<br>• Unable to feed or eat
Respiratory Rate	• Mildly increased	• Respiratory rate in the Yellow Zone	• Respiratory rate in the Red Zone<br>• Decreasing (exhaustion)
Accessory Muscle Use	• None / minimal	• Moderate recession<br>• Tracheal tug<br>• Nasal flaring	• Severe recession<br>• Gasping<br>• Grunting<br>• Extreme pallor<br>• Cyanosis<br>• Absent breath sounds
Apnoeic Episodes	• None	• Abnormal pauses in breathing	• Apnoeic episodes
Oxygen	• No oxygen requirement	• Mild hypoxaemia, corrected by oxygen<br>• Increasing oxygen requirement	• Hypoxaemia, may not be corrected by oxygen

NSW GOVERNMENT | Health

STANDARD PAEDIATRIC OBSERVATION CHART (SPOC)

12 Years and over

☐ Altered Calling Criteria

ALL OBSERVATIONS MUST BE GRAPHED

FAMILY NAME	MRN
GIVEN NAME	☐ MALE ☐ FEMALE
D.O.B. ____/____/____	M.O.
ADDRESS	
LOCATION	

COMPLETE ALL DETAILS OR AFFIX PATIENT LABEL HERE

OTHER CHARTS IN USE

☐ **Fluid Balance** ☐ **Insulin Infusion** ☐ **Other** ______________
☐ **Neurological Observation** ☐ **Pain / Epidural / Patient Control Analgesia** ☐ **Other** ______________
☐ **Neurovascular** ☐ **Resuscitation Plan** ☐ **Other** ______________

PRESCRIBED FREQUENCY OF OBSERVATIONS

Observations must be performed routinely at least 4th hourly, unless advised below

**DATE:**	dd/MM/yy				
**TIME:**	hh:mm				
**Frequency Required**	Twice daily				
**Medical Officer Name** (BLOCK letters)	P. SMITH				
**Medical Officer Signature**	P. SMITH				
**Attending Medical Officer Signature**	R. Bloggs				

ALTERATIONS TO CALLING CRITERIA
MUST BE REVIEWED WITHIN 48 HOURS OR EARLIER IF CLINICALLY INDICATED
Any alterations MUST be signed by a Medical Officer and confirmed by Attending Medical Officer
Document rationale for altering CALLING CRITERIA in the patient's health care record

**DATE:**	dd/MM/yy				
**TIME:**	hh:mm				
**Next review due Date & Time**	dd/MM/yy hh:mm				

Vital Sign	Zone	Standard Thresholds					
**Respiratory Rate**	Yellow Zone	5 - 10<br>30 - 40					
	Red Zone	< 5<br>> 40					
**SpO$_2$**	Yellow Zone	90 - 95					
	Red Zone	< 90					
**Heart Rate**	Yellow Zone	40 - 50<br>130 - 150	xxx-xxx				
	Red Zone	< 40<br>> 150	≤ or ≥ xxx				
**Other**	Yellow Zone						
	Red Zone						
**Medical Officer Name** (BLOCK letters)			P. SMITH				
**Medical Officer Signature**			P. SMITH				
**Attending Medical Officer Signature**			R. Bloggs				

	Date	Time	INTERVENTIONS / COMMENTS / ACTIONS
1.			
2.			
3.			
4.			

EXAMPLE

STANDARD PAEDIATRIC OBSERVATION CHART 12 YEARS AND OVER SMR110.019

REFER TO YOUR LOCAL CLINICAL EMERGENCY RESPONSE SYSTEM (CERS) PROTOCOL FOR INSTRUCTIONS ON HOW TO MAKE A CALL TO ESCALATE CARE FOR YOUR PATIENT

CHECK THE HEALTH CARE RECORD FOR AN END OF LIFE CARE PLAN WHICH MAY ALTER THE MANAGEMENT OF YOUR PATIENT

Blue Zone Response

IF YOUR PATIENT HAS ANY BLUE ZONE OBSERVATIONS YOU MUST

1. Initiate appropriate clinical care
2. Increase the frequency of observations, as indicated by your patient's condition
3. Manage anxiety, pain and review oxygenation in consultation with the **NURSE IN CHARGE**
4. You can make a call to escalate the care of your patient at any time if you are worried or unsure whether to call

Consider the following:

1. What is usual for your patient and are there documented 'ALTERATIONS TO CALLING CRITERIA'?
2. Does the abnormal observation reflect deterioration in your patient?
3. Is there an adverse trend in observations?

Yellow Zone Response

IF YOUR PATIENT HAS ANY YELLOW ZONE OBSERVATIONS OR ADDITIONAL CRITERIA* YOU MUST

1. Initiate appropriate clinical care
2. Repeat and increase the frequency of observations, as indicated by your patient's condition
3. Consult promptly with the **NURSE IN CHARGE** to decide whether a **CLINICAL REVIEW** (or other CERS) call should be made

Consider the following:

- What is usual for your patient and are there documented 'ALTERATIONS TO CALLING CRITERIA'?
- Does the trend in observations suggest deterioration?
- Is there more than one Yellow Zone observation or additional criteria?
- Are you concerned about your patient?

IF A CLINICAL REVIEW IS CALLED:

1. Reassess your patient and escalate according to your local CERS if the call is not attended within 30 minutes or you are becoming more concerned
2. Document an A-G assessment, reason for escalation, treatment and outcome in your patient's health care record
3. Inform the Attending Medical Officer that a call was made as soon as it is practicable

***Additional YELLOW ZONE Criteria**

- Increasing oxygen requirement
- Poor peripheral circulation
- Greater than expected fluid loss
- Reduced urine output or anuria (< 1mL/kg/hr)
- Altered mental state: Agitation, Combative or Inconsolable
- New, increasing or uncontrolled pain
- New onset of fever > 38.5°C
- BGL 2-3mmol/L
- **Concern by you or any staff or family member**

CONSIDER IF YOUR PATIENT'S DETERIORATION COULD BE DUE TO SEPSIS, DEHYDRATION / HYPOVOLAEMIA / HAEMORRHAGE, OR AN OVERDOSE / OVER SEDATION

Red Zone Response

IF YOUR PATIENT HAS ANY RED ZONE OBSERVATIONS OR ADDITIONAL CRITERIA# YOU MUST CALL FOR A RAPID RESPONSE (as per local CERS) AND

1. Initiate appropriate clinical care
2. Inform the **NURSE IN CHARGE** that you have called for a Rapid Response
3. Repeat and increase the frequency of observations, as indicated by your patient's condition
4. Document an A-G assessment, reason for escalation, treatment and outcome in your patient's health care record
5. Inform the Attending Medical Officer that a call was made as soon as it is practicable

#Additional RED ZONE Criteria

- **Cardiac or respiratory arrest**
- **Circulatory collapse**
- **Patient unresponsive**
- **New onset of stridor**
- Deterioration not reversed within 1 hour of Clinical Review
- 3 or more simultaneous 'Yellow Zone' observations
- Significant bleeding
- Sudden decrease in Level of Consciousness (a drop of 2 or more points on the GCS)
- New or prolonged seizure activity
- BGL < 2mmol/L or symptomatic
- Lactate ≥ 4mmol/L
- **Serious concern by you or any staff or family member**

DO NOT PRINT

BINDING MARGIN - NO WRITING

Holes Punched as per AS2828.1: 2012

SMR110019

SMR110018

Holes Punched as per AS2828.1: 2012

BINDING MARGIN - NO WRITING

NSW GOVERNMENT Health

STANDARD PAEDIATRIC OBSERVATION CHART (SPOC)

5 - 11 Years

☐ Altered Calling Criteria

ALL OBSERVATIONS MUST BE GRAPHED

FAMILY NAME	MRN
GIVEN NAME	☐ MALE ☐ FEMALE
D.O.B. ______/______/______	M.O.
ADDRESS	
LOCATION	

COMPLETE ALL DETAILS OR AFFIX PATIENT LABEL HERE

5-11 Years

Section	Parameter	Scale	Date / Time	Scale
AIRWAY / BREATHING	Respiratory Rate ● (breaths per minute)	60		60
		55		55
		50		50
		45		45
		40		40
		35		35
		30		30
		25		25
		20		20
		15		15
		10		10
		5		5
	Respiratory Distress	Severe		Severe
		Moderate		Mod
		Mild		Mild
		Normal		Normal
	SpO_2 % ●	100		100
		95		95
		90		90
		85		85
		80		80
		75		75
		<70		<70
		Probe Change		Probe Change
	Oxygen	L/min or %		L/min or %
		Device		Device
CIRCULATION	Heart Rate ● (beats per minute)	180		180
		170		170
		160		160
		150		150
		140		140
		130		130
		120		120
		110		110
		100		100
		90		90
		80		80
		70		70
		60		60
		50		50
		40		40
	Capillary Refill	≥ 3 Seconds		≥ 3 Seconds
		< 3 Seconds		< 3 Seconds
	Blood Pressure (mmHg) > < SBP is the trigger	160		160
		150		150
		140		140
		130		130
		120		120
		110		110
		100		100
		90		90
		80		80
		70		70
		60		60
		50		50
		40		40
		30		30
		20		20
		10		10
		Initials		Initials

☐ Increase Frequency of Observations ☐ Clinical Review ☐ Rapid Response

NH606543 201213

NSW GOVERNMENT Health

STANDARD PAEDIATRIC OBSERVATION CHART (SPOC)

5 - 11 Years

☐ Altered Calling Criteria

ALL OBSERVATIONS MUST BE GRAPHED

FAMILY NAME	MRN
GIVEN NAME	☐ MALE ☐ FEMALE
D.O.B. ______/______/______	M.O.
ADDRESS	
LOCATION	

COMPLETE ALL DETAILS OR AFFIX PATIENT LABEL HERE

5-11 Years

Section	Parameter	Scale	Date / Time	Scale
DISABILITY	Level of Consciousness	Alert		Alert
		Verbal		Verbal
		Pain		Pain
		Unresponsive		Unresponsive
	Enter appropriate letter. A= Alert, V= Rousable only by voice (consider GCS). P= Rousable only by central pain (conduct GCS). U=Unresponsive			
	Pain Score	Severe (7-10)		Severe (7-10)
		Moderate (4-6)		Moderate (4-6)
		Mild (1-3)		Mild (1-3)
		Nil		Nil
EXPOSURE	Temperature (°C) ● (Check unit policy)	41		41
		40.5		40.5
		40		40
		39.5		39.5
		39		39
		38.5		38.5
		38		38
		37.5		37.5
		37		37
		36.5		36.5
		36		36
		35.5		35.5
		35		35
		34.5		34.5
		34		34
	BGL			BGL
	Weight			Weight
	Initials			Initials

DO NOT PRINT

CONSIDER EARLIER ESCALATION OF PATIENTS WITH

- Chronic or complex conditions
- Post-operative
- Pre-Existing cardiac or respiratory conditions
- Opioid Infusions

ADDITIONAL CRITERIA FOR ESCALATION ON BACK PAGE

ASSESSMENT OF RESPIRATORY DISTRESS

	MILD	MODERATE	SEVERE
Airway	• Stridor on exertion	• Stridor at rest<br>• Partial airway obstruction	• New onset of stridor<br>• Imminent airway obstruction
Behaviour & Feeding	• Normal<br>• Talks in sentences	• Some / intermittent irritability<br>• Difficulty talking or crying<br>• Difficulty feeding or eating	• Agitated / confused<br>• Drowsy<br>• Unable to talk or cry<br>• Unable to feed or eat
Respiratory Rate	• Mildly increased	• Respiratory rate in the Yellow Zone	• Respiratory rate in the Red Zone<br>• Decreasing (exhaustion)
Accessory Muscle Use	• None / minimal	• Moderate recession<br>• Tracheal tug<br>• Nasal flaring	• Severe recession<br>• Gasping<br>• Grunting<br>• Extreme pallor<br>• Cyanosis<br>• Absent breath sounds
Apnoeic Episodes	• None	• Abnormal pauses in breathing	• Apnoeic episodes
Oxygen	• No oxygen requirement	• Mild hypoxaemia, corrected by oxygen<br>• Increasing oxygen requirement	• Hypoxaemia, may not be corrected by oxygen

NSW GOVERNMENT Health

STANDARD PAEDIATRIC OBSERVATION CHART (SPOC)

5 - 11 Years

☐ Altered Calling Criteria

ALL OBSERVATIONS MUST BE GRAPHED

FAMILY NAME	MRN
GIVEN NAME	☐ MALE ☐ FEMALE
D.O.B. _______ / _______ / _______	M.O.
ADDRESS	
LOCATION	

COMPLETE ALL DETAILS OR AFFIX PATIENT LABEL HERE

5-11 Years

OTHER CHARTS IN USE

☐ Fluid Balance ☐ Insulin Infusion ☐ Other ____________

☐ Neurological Observation ☐ Pain / Epidural / Patient Control Analgesia ☐ Other ____________

☐ Neurovascular ☐ Resuscitation Plan ☐ Other ____________

PRESCRIBED FREQUENCY OF OBSERVATIONS

Observations must be performed routinely at least 4th hourly, unless advised below

**DATE:**	dd/MM/yy				
**TIME:**	hh:mm				
**Frequency Required**	Twice daily				
**Medical Officer Name** (BLOCK letters)	P. SMITH				
**Medical Officer Signature**	P. SMITH				
**Attending Medical Officer Signature**	R. Bloggs				

EXAMPLE

ALTERATIONS TO CALLING CRITERIA

MUST BE REVIEWED WITHIN 48 HOURS OR EARLIER IF CLINICALLY INDICATED

Any alterations MUST be signed by a Medical Officer and confirmed by Attending Medical Officer

Document rationale for altering CALLING CRITERIA in the patient's health care record

		**DATE:**	dd/MM/yy				
		**TIME:**	hh:mm				
		**Next review due Date & Time**	dd/MM/yy hh:mm				
**Vital Sign**	**Zone**	**Standard Thresholds**					
**Respiratory Rate**	Yellow Zone	10 - 15<br>35 - 50					
	Red Zone	<10<br>>50					
**SpO_2**	Yellow Zone	90 - 95					
	Red Zone	<90					
**Heart Rate**	Yellow Zone	60 - 70<br>140 - 160	xxx-xxx				
	Red Zone	<60<br>>160	≤ or ≥ xxx				
**Other**	Yellow Zone						
	Red Zone						
		**Medical Officer Name** (BLOCK letters)	P. SMITH				
		**Medical Officer Signature**	P. SMITH				
		**Attending Medical Officer Signature**	R. Bloggs				

EXAMPLE

	Date	Time	INTERVENTIONS / COMMENTS / ACTIONS
1.			
2.			
3.			
4.			

REFER TO YOUR LOCAL CLINICAL EMERGENCY RESPONSE SYSTEM (CERS) PROTOCOL FOR INSTRUCTIONS ON HOW TO MAKE A CALL TO ESCALATE CARE FOR YOUR PATIENT

CHECK THE HEALTH CARE RECORD FOR AN END OF LIFE CARE PLAN WHICH MAY ALTER THE MANAGEMENT OF YOUR PATIENT

Blue Zone Response

IF YOUR PATIENT HAS ANY BLUE ZONE OBSERVATIONS YOU MUST

1. Initiate appropriate clinical care
2. Increase the frequency of observations, as indicated by your patient's condition
3. Manage anxiety, pain and review oxygenation in consultation with the **NURSE IN CHARGE**
4. You can make a call to escalate the care of your patient at any time if you are worried or unsure whether to call

Consider the following:

1. What is usual for your patient and are there documented 'ALTERATIONS TO CALLING CRITERIA'?
2. Does the abnormal observation reflect deterioration in your patient?
3. Is there an adverse trend in observations?

Yellow Zone Response

IF YOUR PATIENT HAS ANY YELLOW ZONE OBSERVATIONS OR ADDITIONAL CRITERIA✱ YOU MUST

1. Initiate appropriate clinical care
2. Repeat and increase the frequency of observations, as indicated by your patient's condition
3. Consult promptly with the **NURSE IN CHARGE** to decide whether a **CLINICAL REVIEW** (or other CERS) call should be made

Consider the following:

- What is usual for your patient and are there documented 'ALTERATIONS TO CALLING CRITERIA'?
- Does the trend in observations suggest deterioration?
- Is there more than one Yellow Zone observation or additional criteria?
- Are you concerned about your patient?

IF A CLINICAL REVIEW IS CALLED:

1. Reassess your patient and escalate according to your local CERS if the call is not attended within 30 minutes or you are becoming more concerned
2. Document an A-G assessment, reason for escalation, treatment and outcome in your patient's health care record
3. Inform the Attending Medical Officer that a call was made as soon as it is practicable

✱Additional YELLOW ZONE Criteria

- Increasing oxygen requirement
- Poor peripheral circulation
- Greater than expected fluid loss
- Reduced urine output or anuria (< 1mL/kg/hr)
- Altered mental state: Agitation, Combative or Inconsolable
- New, increasing or uncontrolled pain
- New onset of fever > 38.5°C
- BGL 2-3mmol/L
- **Concern by you or any staff or family member**

CONSIDER IF YOUR PATIENT'S DETERIORATION COULD BE DUE TO SEPSIS, DEHYDRATION / HYPOVOLAEMIA / HAEMORRHAGE, OR AN OVERDOSE / OVER SEDATION

Red Zone Response

IF YOUR PATIENT HAS ANY RED ZONE OBSERVATIONS OR ADDITIONAL CRITERIA# YOU MUST CALL FOR A RAPID RESPONSE (as per local CERS) AND

1. Initiate appropriate clinical care
2. Inform the **NURSE IN CHARGE** that you have called for a Rapid Response
3. Repeat and increase the frequency of observations, as indicated by your patient's condition
4. Document an A-G assessment, reason for escalation, treatment and outcome in your patient's health care record
5. Inform the Attending Medical Officer that a call was made as soon as it is practicable

#Additional RED ZONE Criteria

- **Cardiac or respiratory arrest**
- **Circulatory collapse**
- **Patient unresponsive**
- **New onset of stridor**
- Deterioration not reversed within 1 hour of Clinical Review
- 3 or more simultaneous 'Yellow Zone' observations
- Significant bleeding
- Sudden decrease in Level of Consciousness (a drop of 2 or more points on the GCS)
- New or prolonged seizure activity
- BGL < 2mmol/L or symptomatic
- Lactate ≥ 4mmol/L
- **Serious concern by you or any staff or family member**

DO NOT PRINT

BINDING MARGIN - NO WRITING

Holes Punched as per AS2828.1: 2012

SMR110018
